# Pathogen Identification Direct From Polymicrobial Specimens Using Membrane Glycolipids

**DOI:** 10.1038/s41598-018-33681-8

**Published:** 2018-10-26

**Authors:** William E. Fondrie, Tao Liang, Benjamin L. Oyler, Lisa M. Leung, Robert K. Ernst, Dudley K. Strickland, David R. Goodlett

**Affiliations:** 1 Center for Vascular and Inflammatory Diseases, University of Maryland School of Medicine, Baltimore, MD 21201 USA; 2 Department of Pharmaceutical Sciences, University of Maryland School of Pharmacy, Baltimore, MD 21201 USA; 3 Toxicology and Pharmacology, University of Maryland School of Medicine, Baltimore, MD 21201 USA; 4 Department of Microbial Pathogenesis, University of Maryland School of Dentistry, Baltimore, MD 21201 USA; 5 Present Address: Divisions of Microbiology and Molecular Biology, Laboratories Administration, Maryland Department of Health, Baltimore, Maryland 21205 USA; 6 Department of Surgery, University of Maryland School of Medicine, Baltimore, MD 21201 USA; 7 Department of Physiology, University of Maryland School of Medicine, Baltimore, MD 21201 USA

## Abstract

With the increased prevalence of multidrug-resistant Gram-negative bacteria, the use of colistin and other last-line antimicrobials is being revisited clinically. As a result, there has been an emergence of colistin-resistant bacterial species, including *Acinetobacter baumannii* and *Klebsiella pneumoniae*. The rapid identification of such pathogens is vitally important for the effective treatment of patients. We previously demonstrated that mass spectrometry of bacterial glycolipids has the capacity to identify and detect colistin resistance in a variety of bacterial species. In this study, we present a machine learning paradigm that is capable of identifying *A. baumannii*, *K. pneumoniae* and their colistin-resistant forms using a manually curated dataset of lipid mass spectra from 48 additional Gram-positive and -negative organisms. We demonstrate that these classifiers detect *A. baumannii* and *K. pneumoniae* in isolate and polymicrobial specimens, establishing a framework to translate glycolipid mass spectra into pathogen identifications.

## Introduction

The rapid identification and characterization of pathogens in an infection is critical to inform treatment decisions and improve patient outcome. The detection of antimicrobial-resistant pathogens has become increasingly important due to the growing prevalence of antimicrobial-resistant isolates^1^. The current standard for pathogen identification and characterization in clinical laboratories incorporates morphological and biochemical methods, which are often slow to perform and yield incomplete diagnoses^2^. Matrix-assisted laser desorption/ionization time-of-flight mass spectrometry (MALDI-TOF MS) of protein fingerprints has gained popularity as the predominant method for pathogen identification with the FDA-approved implementations of the Bruker MALDI Biotyper and the bioMérieux VITEK MS systems^3–6^. Though robust and facile in comparison to the traditional methods, these MALDI-TOF MS platforms still suffer from the need for prior cell culture to obtain pure colonies and cannot distinguish organisms in a polymicrobial infection or direct from biological samples, such as blood, urine, or wound effluent. Additionally, detection of antimicrobial resistance is currently unavailable on the FDA-approved platforms, though β-lactamase detection is implemented on the Biotyper research-only platform. In an effort to develop a complementary method to the protein-based MALDI-TOF MS strategies, we previously demonstrated the use of microbial membrane glycolipids as analytes for MALDI-TOF MS identification of pathogens, which extends a long line of work identifying bacteria by their respective lipid profiles^7–10^.

Microbial membranes are composed, in part, of complex glycolipids that are present in high abundance. In Gram-negative bacteria, the major glycolipid constituent of the outer membrane’s outer leaflet is lipopolysaccharide (LPS)^11^. For *E. coli*, these glycolipids have been estimated at 10^6^ copies per bacterium^12^. Previous studies have demonstrated diversity in the structure of LPS across bacterial species including the LPS membrane anchor component, lipid A^13^. This LPS component comprises a diglucosamine backbone substituted with fatty acyl chains and terminal phosphate residues. The structural diversity of lipid A has been observed in the species-specific composition of fatty acyl chains and phosphate modifications that result in unique mass spectral profiles^13,14^. Additionally, we and others have described lipid A modifications to the terminal phosphates that occur with antimicrobial resistance, which include phosphoethanolamine and aminoarabinose additions^15–20^. With analogous membrane lipids present in Gram-positive bacterial membranes, such as lipoteichoic acid, and ubiquitous lipids like cardiolipin, high mass lipids are useful for the identification of virtually all bacterial species by mass spectrometry^21^.

Our previous work found that pathogens are distinguishable by MALDI-TOF MS of membrane glycolipids. This work generated a glycolipid mass spectral dataset containing 2068 mass spectra of intact molecular ions from 50 microbial species^7^. One notable advantage of our glycolipid-based approach over the popular protein-based method is that culture can be circumvented and polymicrobial infections detected. In this study, we sought to utilize this published dataset to develop generalizable methods for bacterial species identification and detection of antimicrobial resistance through robust feature extraction and machine learning. Furthermore, we aimed to evaluate the potential of using glycolipid MALDI-TOF mass spectra to identify pathogens directly from polymicrobial infections in urine. These complex infections are often difficult to treat and characterize, and generally result in increased risk for the patient^22–24^.

We selected two prototypical organisms as targets for these tasks: *Acinetobacter baumannii* and *Klebsiella pneumoniae*. These pathogens account for a high incidence of hospital-acquired infections, resulting in increased morbidity in hospitalized patients, especially those who are immunocompromised. Furthermore, both are frequently observed with multi-drug resistance phenotypes, thereby increasing reliance on the cationic antimicrobial peptide, colistin, as a last-line therapeutic. However, the prevalence of colistin resistance in these pathogens and others has grown over recent years, indicating a need to rapidly discriminate between colistin-susceptible and -resistant strains^19,20^. Using the glycolipid mass spectral dataset presented in Leung *et al*., we trained machine learning classifiers to identify *A. baumannii* and *K. pneumoniae* mass spectra from the library and detect profiles corresponding to colistin resistance with high confidence^7^. With these classifiers, we were then able to identify *A. baumannii* and *K. pneumoniae* from simulated polymicrobial glycolipid mass spectra and a small set of *in vitro* models representing polymicrobial urinary tract infections (UTIs). These results present a viable machine learning approach to microbial identification from glycolipid mass spectra and suggest that these will be useful for identification directly from polymicrobial samples.

## Results

### The dataset of isolate glycolipid mass spectra

**Figure 1:**
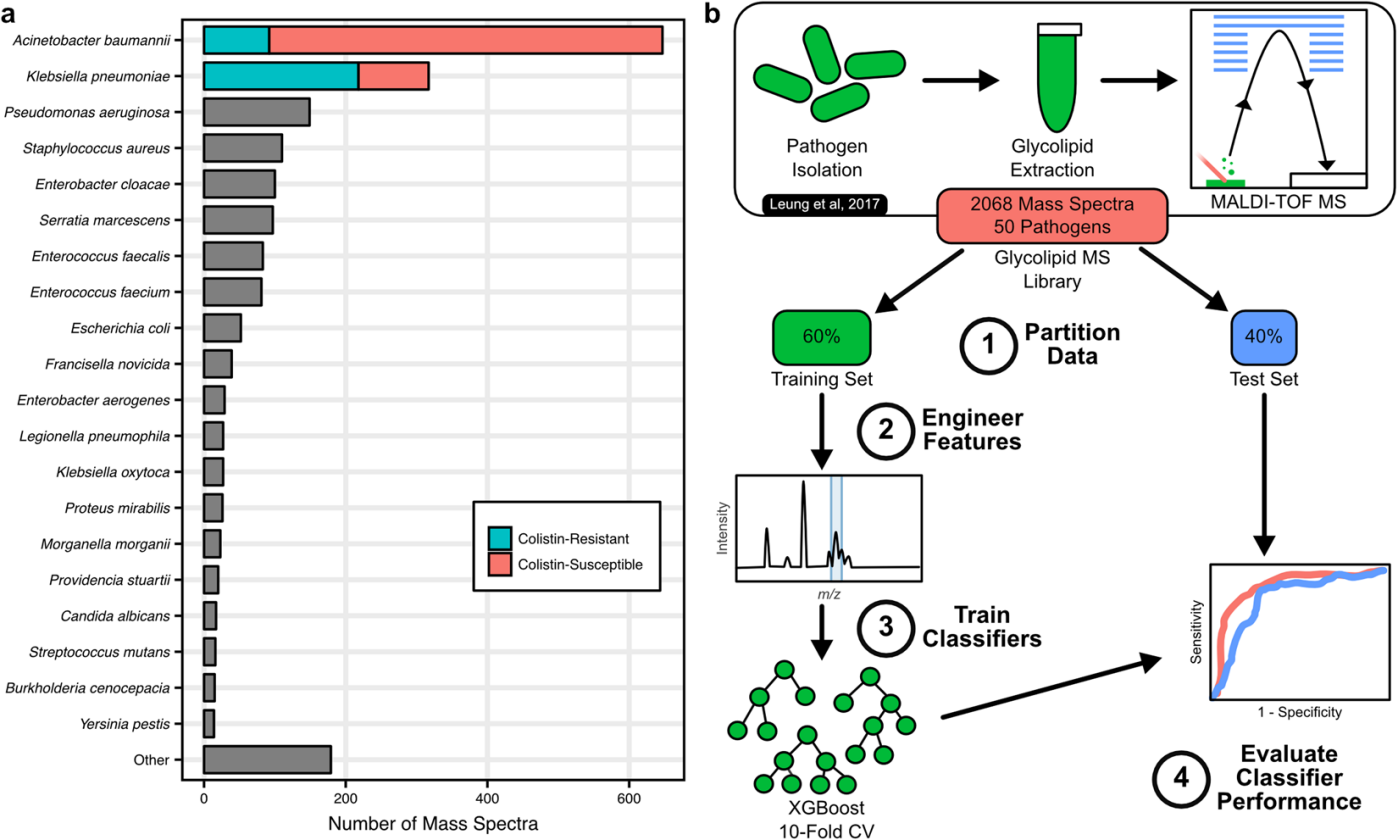
The glycolipid library is used to train classifiers for *A. baumannii* and *K. pneumoniae*. (**a**) The number of mass spectra for top species contained in the isolate glycolipid mass spectral dataset. The colistin-resistant (blue) and colistin-susceptible (red) for *A. baumannii* and *K. pneumoniae* mass spectra were selected as targets for machine learning due to the considerable number of mass spectra for each. (**b**) The workflow for classifier training and evaluation from the isolate glycolipid dataset is outlined.

The intact glycolipid mass spectral dataset presented in Leung *et al*. served as the dataset for training machine learning classifiers to identify *A. baumannii* and *K. pneumoniae* mass spectra from the other microbial species and further discriminate colistin-resistant from colistin-susceptible strains^7^. The mass spectra in the dataset were generated by MALDI-TOF MS analysis of glycolipid extracts from isolates grown in liquid culture, resulting in mass spectra of intact molecular ions. In total, this dataset contains 2068 mass spectra from 50 unique microbial species (Fig. 1a). Included in this dataset were technical and biological replicates from one or more strain of each microbial species. Importantly for our classifiers, a large proportion of these mass spectra were generated from low passage clinical isolates from *A. baumannii* (647 mass spectra from 213 isolates) and *K. pneumoniae* (317 mass spectra from 60 isolates). This dataset encompasses various levels of biological and technical variability for the library species and provided a suitable training set for classifiers targeting these *A. baumannii* and *K. pneumoniae*.

### Development of a machine learning paradigm for bacterial identification from glycolipid mass spectra

Using the Leung *et al*. glycolipid mass spectral dataset, we sought to develop methods for training classifiers that could be extended for new species and would be generalizable to the task of identifying organisms from polymicrobial mixtures in a single mass spectrum (Fig. 1b)^7^. Initially, the library was partitioned into a randomized training set (60% of the mass spectra) and a test set (40% of the mass spectra), independently for the *A. baumannii* and *K. pneumoniae* classifiers. The training set underwent automated feature selection for each target organism, which is detailed below. The extracted features were then used to train a gradient boosted tree model, utilizing the XGBoost algorithm for each target organism^25^. For *A. baumannii* and *K. pneumoniae*, classifiers were trained to identify mass spectra containing colistin-resistant isolates, colistin-susceptible isolates, or no isolate of the target species. In addition to these XGBoost classifiers, baseline classifiers were created using the intensity from single features corresponding to the most prominent species-specific and resistance-associated ions for *A. baumannii* (*m/z* 1910 for species and *m/z* 2033 for resistance) and *K. pneumoniae* (*m/z* 1840 for species and *m/z* 1971 for resistance)^7,15–17^. These Single Feature baseline classifiers provided a baseline to define the performance of the machine learning strategy. The performances of all resulting models were then evaluated using the test set; investigating metrics such as accuracy, sensitivity, and specificity, in addition to the receiver operating characteristic (ROC) and precision-recall (PR) curves.

### Feature engineering and automated feature extraction

**Figure 2:**
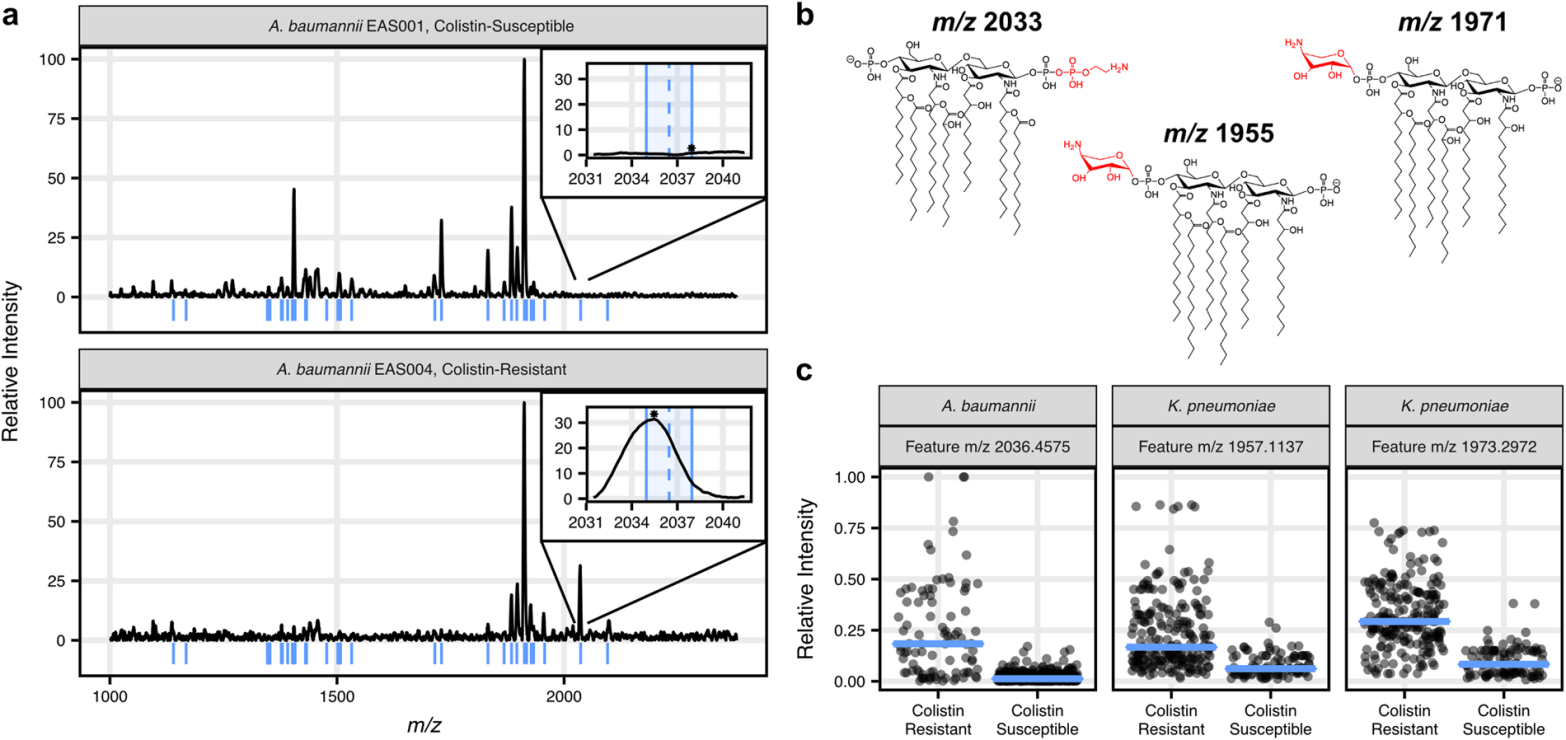
Feature engineering extracts known ions associated with colistin resistance. (**a**) Example mass spectra for colistin-susceptible (top) and colistin-resistant *A. baumannii* (bottom) are shown. The blue lines beneath each spectrum indicate the median *m/z* of the features chosen for the *A. baumannii* classifier. The inset panels show the ±1.5 *m/z* feature extraction window for a resistance-associated ion with a median *m/z* 2036.4575 (blue dashed line). The local maximum intensity is indicated with an asterisk. (**b**) Specific lipid A structures are associated with colistin resistance in *A. baumannii* (*m/z* 2033) and *K. pneumoniae* (*m/z* 1955 and *m/z* 1971). (**c**) To verify feature extraction performed as expected, the distributions of features indicative of resistance-associated ions were compared between colistin-resistant and -susceptible mass spectra for each species. The median is shown as a blue line. The higher intensities of resistance-associated ions in colistin-resistant mass spectra indicated that the feature extraction method performed well.

For feature engineering, we chose an approach that would be extendable from the isolate mass spectra in the dataset to mass spectra containing multiple species, representing a polymicrobial infection. To this end, the top 50 average most intense ions from the target organisms were identified from mass spectra in the training set and selected as feature ions for extraction (Fig. 2a). Smoothing is required due to the variable mass accuracy and resolution of these mass spectra, resulting in feature ions correlated to the average *m/*z of each molecular ion. To extract these features from each mass spectrum in the dataset, the local maximum intensity within a 3 *m/z* window centered on each feature ion was extracted (Fig. 2a, inset). As a result, these features are centered at *m/z* values greater than the monoisotopic mass of the molecular ion and may be further skewed by additional ions in the region. The extracted features were then normalized relative to the intensity of the most intense feature. This simple extraction method resulted in features that were selected based on their presence in the mass spectra of the target organism and independent of noise from irrelevant regions of the mass spectra, such as ions contributed from other organisms. Though more features could have been selected, we found 50 features to be more than adequate for identification, which is supported by the relative scarcity of ions in the glycolipid mass spectra as compared to those obtained by protein fingerprinting. However, for mass spectra collected with higher resolution, more features likely would be useful. The features were named by the *m/z* center of the extraction window, reported to beyond analytical significance. This ensured unambiguous feature names in the event that automatically selected features occupy directly adjacent *m/z* windows.

To verify that feature extraction functioned as expected, we investigated the extracted features corresponding to known monoisotopic ions associated with a colistin resistance phenotype. These ions were *m/z* 2033 (feature *m/z* 2036.4575) for *A. baumannii* and *m/z* 1955 and *m/z* 1971 for *K. pneumoniae* (features *m/z* 1957.1469 and *m/z* 1973.2972, respectively)^17,19,20^. Previous studies have identified these ions as lipid A structures with terminal phosphates that have been modified with phosphoethanolamine (+*m/z* 123 shift from ion at *m/z* 1910) and aminoarabinose (+*m/z* 131 shift from ions at *m/z* 1824 and *m/*z 1840) additions for *A. baumannii* and *K. pneumoniae*, respectively (Fig. 2b)^7,15–17^. As predicted, extracted features corresponding to these resistance-associated structures are elevated in the glycolipid mass spectra originating from the colistin-resistant strains (Fig. 2c).

### Classifier performance on glycolipid dataset mass spectra

**Figure 3:**
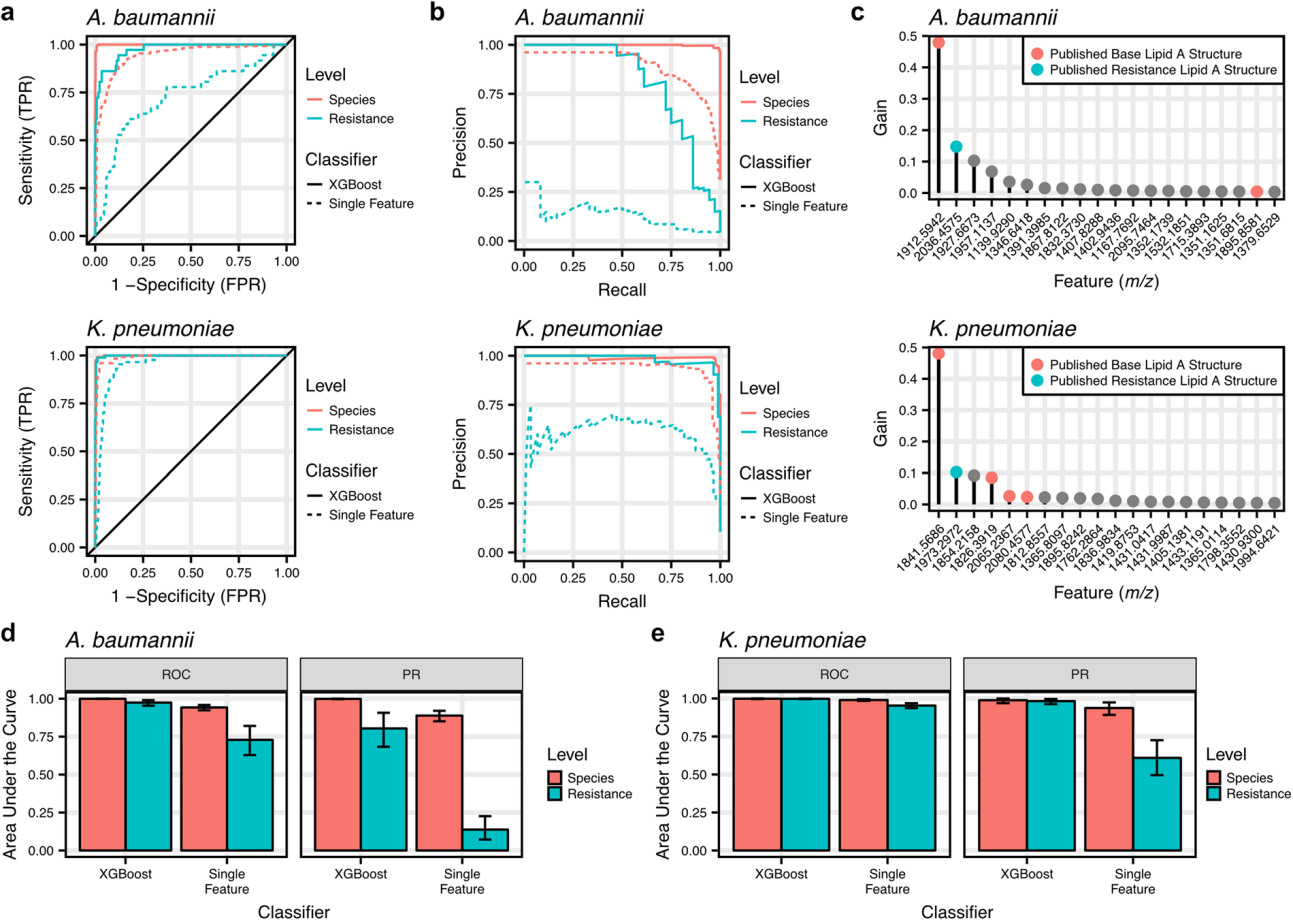
The classifiers distinguish species and colistin resistance for *A. baumannii* and *K. pneumoniae*. (**a**) Receiver operating characteristic (ROC) curves were created from the trained 3-class models to evaluate species and colistin resistance identification for the XGBoost classifiers and the baseline Single Feature classifiers using one-vs-all methods. These revealed consistently high sensitivity and specificity for both XGBoost classifiers. (**b**) Similarly, precision-recall (PR) curves were created to evaluate the overall precision of the classifiers for each target class. **(c)** Gain, a measure of feature importance in the classifier, was investigated for the features in the *A. baumannii* and *K. pneumoniae* classifiers. Features representing previously published species-specific lipid A ions appeared to be the most important features for classification^19,20^. (**d**,**e**) The area under the curve (AUC) for ROC and PR curves demonstrated superior performance of the XGBoost classifiers over the baseline Single Feature classifiers. Additionally, the consistently high AUC for both species and colistin resistance indicate overall high performance of these classifiers. Error bars indicate 95% confidence intervals.

The trained XGBoost classifiers and Single Feature baseline classifiers were evaluated using the test set, which contained 40% of the glycolipid library and was held out from classifier training (Supplementary Data S1). Analysis of the ROC curves suggested that the trained XGBoost classifiers reliably identified both *A. baumannii* and *K. pneumoniae* with species level areas under the curve (AUCs) of 0.999 (0.998 to 1.000) and 0.999 (0.996 to 1.000), respectively (Fig. 3a,d and e). Additionally, the ROC curves indicated that the XGBoost classifiers can distinguish colistin-resistant and -susceptible isolates of both target species from each other and from the other species in the library. We also investigated the PR curves of the classifiers, which indicated high performance at species-level identifications and detection of colistin resistance with all AUCs above 0.80 for the XGBoost classifiers (Fig. 3b,d and e). The XGBoost classifiers markedly outperformed the Single Feature baseline classifiers in all of these metrics.

To determine if the models agree with prior knowledge of species- and resistance-specific lipid A structures, the classifiers were investigated for the importance of each feature in classification. The feature importance metric, gain, is a measure of the relative improvement in classification accuracy when the feature is considered. As expected, many of the most important features for successful classification corresponded to known lipid A structures (Fig. 3c). For *A. baumannii*, the most important feature is centered at *m/z* 1913 and corresponds to the commonly observed hepta-acylated lipid A structure for the species with monoisotopic *m/z* 1910. Additionally, the second most important feature corresponds to the known phosphoethanolamine-modified lipid A structure at monoisotopic *m/z* 2033, which is associated with colistin resistance. This trend holds for the *K. pneumoniae* classifier as well, with both the base and colistin resistance-associated structures appearing among the most important features.

A failure to detect colistin resistance in a clinical diagnosis results in the loss of time for efficacious patient treatment and the potential for spreading of resistant strains. Due to these consequences, we chose to evaluate the classifiers at a high threshold requiring 97% sensitivity, which allows for one missed sample in the least prevalent class. When this threshold was imposed, the XGBoost classifiers maintained low false positive rates (Supplementary Fig. S1), in comparison to the large number of false positives obtained by the Single Feature baseline classifiers. Further evaluation of the XGBoost classifiers at this high sensitivity demonstrated accuracy and specificity for both species and determining colistin-resistance (Supplementary Fig. S1). However, the colistin-resistant classifiers do suffer from an increased false discovery rate at the rigorous 97% sensitivity threshold, as indicated by the decreased precision. In particular, the detection of *A. baumannii* colistin resistance appeared to suffer most, which we partially attribute to the reliance on a single resistance ion for identification.

### Simulation of polymicrobial glycolipid mass spectra

In order to rapidly identify infections directly from biological samples, future classifiers may need to make inferences from mass spectra containing mixtures of organisms, like those found in polymicrobial infections. With the current performance of the classifiers on the isolate species mass spectra in our glycolipid dataset, we next sought to determine how our current classifiers would perform in a polymicrobial infection model. To this aim, we combined glycolipid mass spectra from the isolate dataset to simulate mixtures of species in single mass spectra.

Polymicrobial mass spectra were simulated by selecting combinations of collected mass spectra from individual species and combined as a weighted average. Between two and five species were randomly selected for a mass spectrum, with an increased probability of containing mass spectral features from *A. baumannii* or *K. pneumoniae*. Additionally, half of the mass spectra containing either *A. baumannii* or *K. pneumoniae* were from a colistin-resistant isolate. Coefficient weights were randomly drawn from 1, 0.5, 0.25, and 0.1, such that the simulated mass spectra contained a variety of organisms and weights. In total, we simulated 4,000 polymicrobial mass spectra (Supplementary Data S2). Additionally, 1,000 mass spectra of individual species were generated using the same simulation procedure to provide a baseline for comparison and ensure the fidelity of the simulation process.

**Figure 4:**
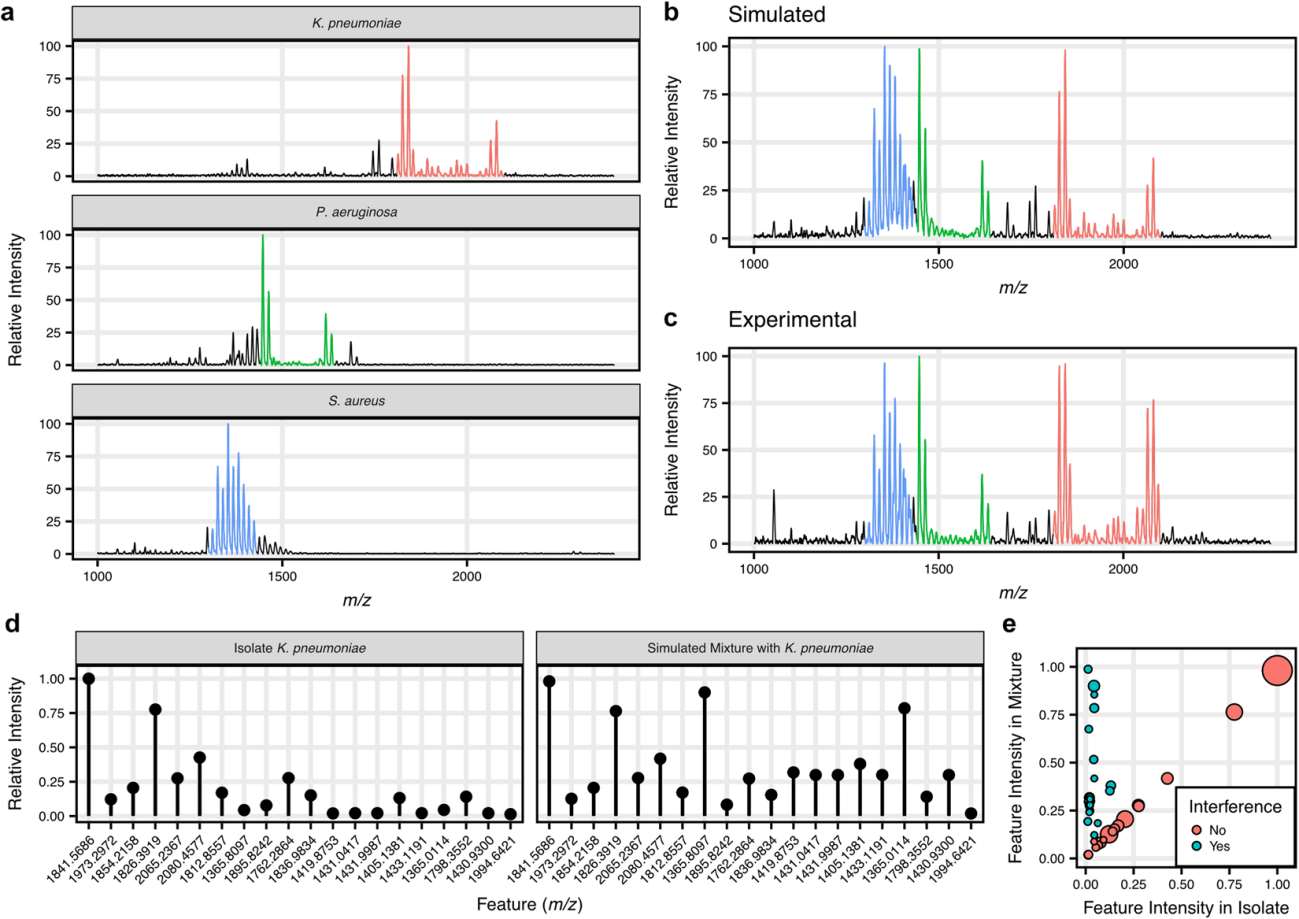
Simulated polymicrobial mixture glycolipid mass spectra are representative of experimentally derived glycolipid mass spectra from mixtures of organisms. (**a**) Representative glycolipid mass spectra from *K. pneumoniae*, *P. aeruginosa* and *S. aureus* isolates, which are microbial species commonly found in wound effluent together. (**b**) A simulated 1:1:1 mixture of the isolate mass spectra was created as the average of the three isolate mass spectra shown in (**a**). (**c**) An experimentally-derived mixture spectrum from Leung *et al*. was created by performing glycolipid extractions on isolates of the three species and mixing prior to acquisition of the mass spectrum^7^. The simulated polymicrobial mass spectrum appears qualitatively similar to the experimental mass spectrum. (**d**) Feature extraction was performed on the isolate *K. pneumoniae* mass spectrum and the simulated mixture mass spectrum. The extracted intensities are shown for the top 20 features, in order of feature importance. (**e**) To assess how mixtures might affect the extracted features, the extracted intensities for features from the isolate and simulated polymicrobial mass spectra were plotted against each other. The size of the points indicates the importance of each feature in the classifier. Interference was detected for some features (deviance >0.05 from isolate mass spectrum), however the most important features for classification appeared unimpaired.

To verify that our simulated polymicrobial mass spectra were an accurate representation of what would be obtained experimentally, we reproduced the experimental mixture extracted from *K. pneumoniae, P. aeruginosa, and S. aureus* presented in Leung *et al*.^7^. These represent species that have been commonly co-isolated in effluent from polymicrobial infections^26^. Figure 4a displays the experimentally obtained glycolipid mass spectra from individual species, with *m/z* regions containing species-specific ions colored for visibility. The simulated mixture mass spectrum shown in Fig. 4b represents a 1:1:1 simulated mixture of extracts from these three species. A qualitative comparison of the simulated mass spectrum to the experimentally obtained mixture mass spectrum (Fig. 4c) reveals that the simulated mass spectrum accurately reflects the mass spectrum of the experimental mixture. Additionally, we compared the 20 most important features from the *K. pneumoniae* classifier between the isolate mass spectrum and the simulated polymicrobial mass spectrum to verify that feature extraction would perform similarly on polymicrobial mass spectra as on the isolate mass spectra (Fig. 4d). When feature intensities are compared between the simulated polymicrobial spectrum and the isolate *K. pneumoniae* spectrum, we observed that, though some features show variation due to interference (defined as a relative intensity deviation greater than 0.05), many of the most important features remained unchanged (Fig. 4e).

**Figure 5:**
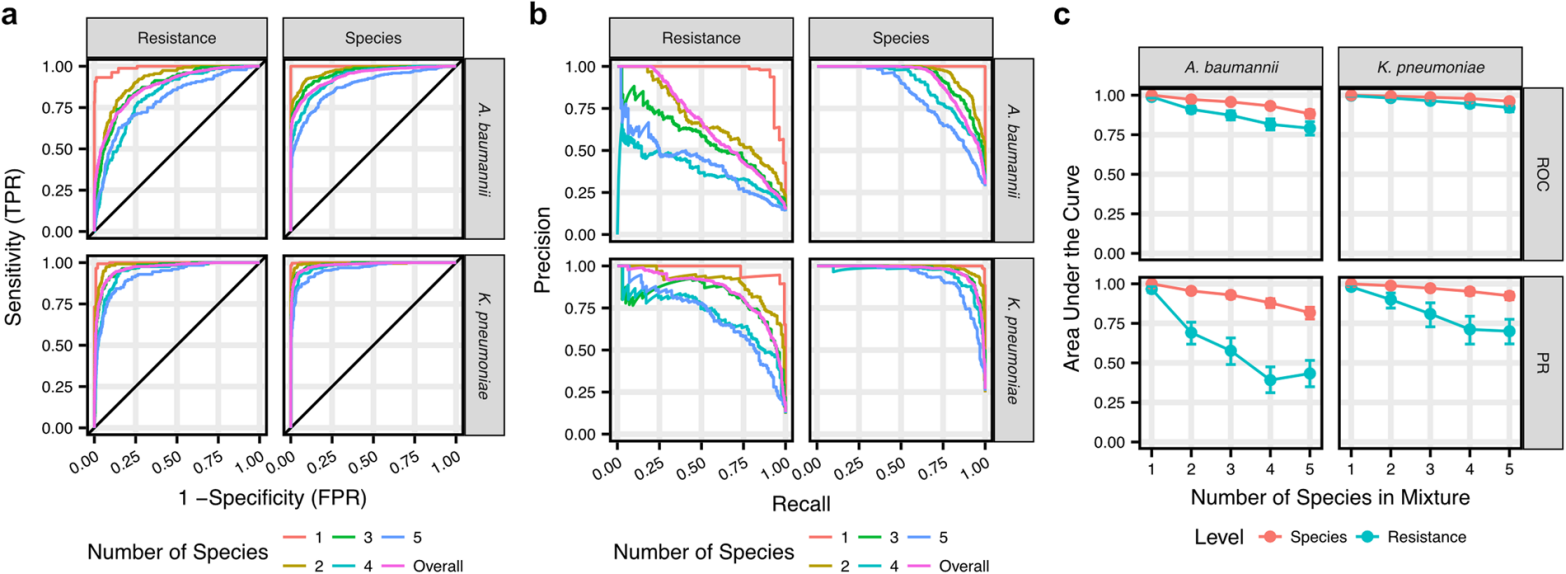
The classifiers detect *A. baumannii* and *K. pneumoniae* species and colistin resistance in simulated polymicrobial mass spectra. (**a**) ROC curves were calculated for the classifiers to evaluate species and colistin resistance detection from the simulated polymicrobial mass spectra using one-vs-all methods and were stratified by the number of species in each mixture. (**b**) Similarly, PR curves were calculated to evaluate overall classifier precision on the simulated polymicrobial mass spectra. (**c**) Investigation of the AUC of the ROC and PR curves stratified by the number of species in each mixture reveals that, while high sensitivity and specificity can be maintained, precision decreases with increasing numbers of species represented in a mass spectrum. Error bars indicate 95% confidence intervals.

Finally, we proceeded to test our XGBoost classifiers on the 4,000 simulated polymicrobial mass spectra. Investigation of the ROC curves revealed sustained classifier performance, even with up to 5 species in the mass spectrum (Fig. 5a and c). However, the PR curves were more revealing for the colistin resistance classifiers, indicating decreased precision as the mass spectrum becomes more complex (Fig. 5b and c). When subjected to the same score threshold that maintained 97% sensitivity for the overall classification of the simulated mass spectra, we observed that the classifiers perform well, especially for species-level identification (Supplementary Fig. S2). However, increased numbers of false discoveries were detected at this rigorous threshold, particularly when attempting to detect colistin resistance, as indicated by the lower precision.

### Classifier performance on *in vitro* polymicrobial UTI models

**Figure 6:**
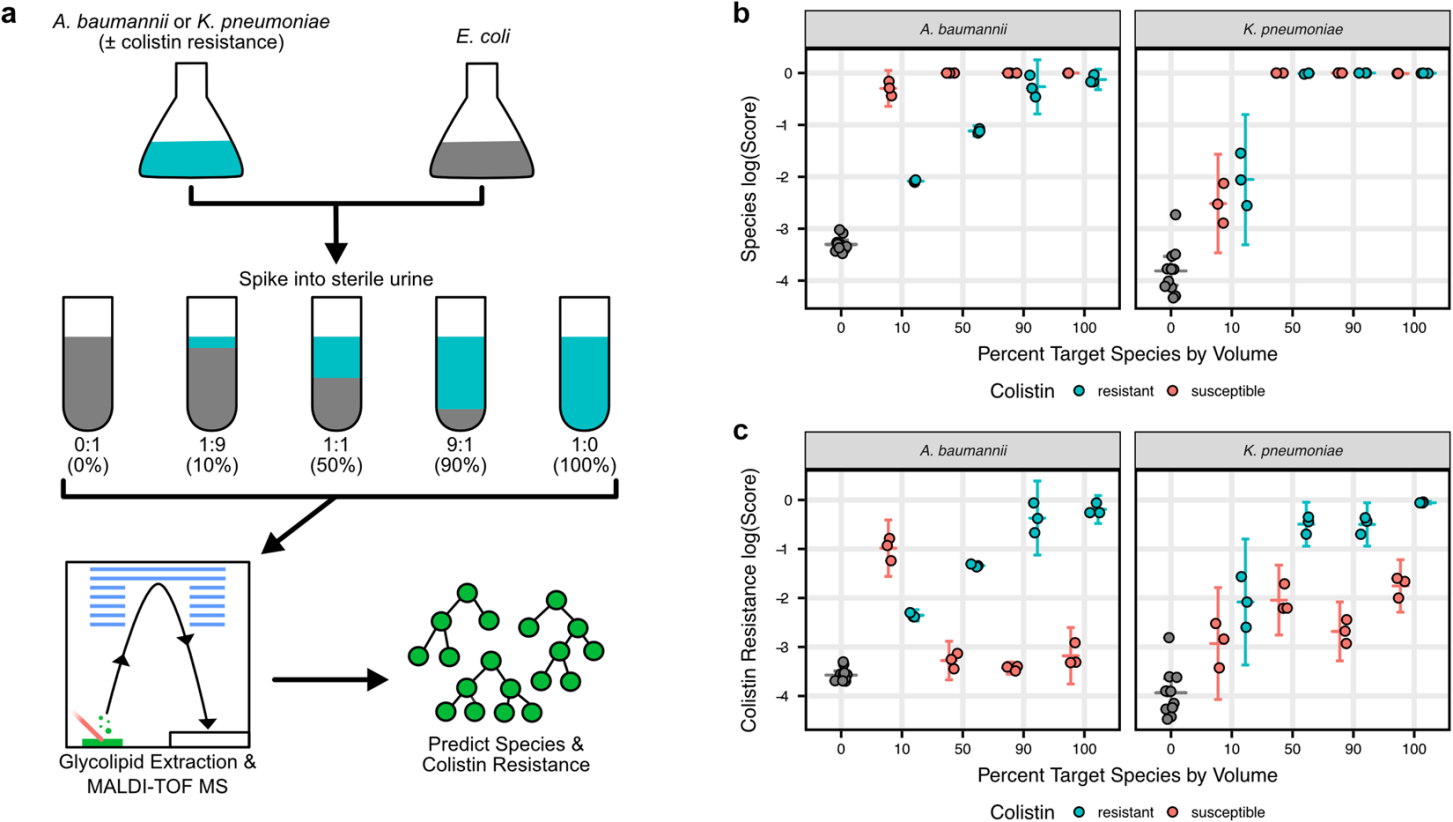
The classifiers detected *A. baumannii* and *K. pneumoniae* species and colistin resistance in UTI spike-in models. (**a**) To assess the performance of the classifiers in polymicrobial specimens and their dependence on species relative abundance, colistin-resistant or -susceptible *A. baumannii* or *K. pneumoniae* were grown as isolates in liquid culture, then mixed with *E. coli* at known volumetric ratios in sterile urine. This yielded mixtures that span a wide spectrum of possible relative species abundances. Glycolipids were then extracted from each sample and analyzed by MALDI-TOF MS. The spectra were then tested with the trained classifiers to detect *A. baumannii*, *K. pneumoniae* and colistin-resistant strains of each. **(b)** High species-level scores were obtained for both target species at ratios greater than or equal to 1:1 (50%) target species to *E. coli*. **(c)** High scores for colistin resistance were obtained for both target species at the same higher ratios as species-level detection, though scores show increased dependence on the proportion of the target organism. Error bars indicate 95% confidence intervals calculated using a t-distribution.

With the current classifier performance on the simulated polymicrobial mass spectra, we created a set of controlled, experimental polymicrobial samples on which to test the *A. baumannii* and *K. pneumoniae* classifiers. In UTIs, *Escherichia coli* is the most commonly identified pathogen, followed distantly by *K. pneumoniae*^27^. However, polymicrobial UTIs containing *E. coli* and *K. pneumoniae* have been previously observed^28^. To create potential polymicrobial UTI samples, we spiked *E. coli* and *K. pneumoniae* at known volumetric ratios into sterile urine (Fig. 6a). Additionally, we created similar mixtures of *E. coli* with *A. baumannii* in sterile urine, although *A. baumannii* is not as common in UTIs. Glycolipids from each sample were then extracted and analyzed by MALDI-TOF MS in triplicate, which revealed ratio-dependent changes in the relative intensities of *E. coli* and target species ions (Supplementary Fig. S3). This presented a challenging test for the *A. baumannii* and *K. pneumoniae* classifiers, with *E. coli* lipid A ions sharing a similar *m/z* range as many of the target species ions.

The presence of *A. baumannii* and *K. pneumoniae*, as well as colistin resistance for each species was then predicted for each of the UTI spike-in samples using the XGBoost classifiers (Supplementary Data S3). In these two-species mixtures, the classifiers were able to reliably identify the target species and resistance in samples with 1:1 or greater ratio of the target organism to *E. coli* (Fig. 6b and c). Many of the scores obtained from 1:1 or greater mixtures are similar to those obtained for classification of the isolate mass spectra. In the case of either target species, the species-level and resistance-level scores at all ratios were decidedly greater than scores for mass spectra containing only *E. coli*. Interestingly, colistin-susceptible *K. pneumoniae* scores rise with increasing ratios in the colistin-resistance classifier. However, we attribute this to the trace abundance of ions in the resistance ion features that are consistently observed even in susceptible strains. These results provide insight into the sensitivity of such classifiers from polymicrobial infections and suggest that they may be useful for species-level identification and antimicrobial resistance screening over the course of diagnosis and treatment.

## Discussion

Infectious diseases pose considerable health and financial burdens worldwide. However, traditional biochemical diagnostics for these pathogens typically require days to perform^2^. This delay can have serious consequences for the treatment of an infection, highlighting the need for methods of rapid and accurate microbial identification. Recently, the suitability of MALDI-TOF MS of bacterial membrane glycolipids, as a novel method for bacterial identification, has been explored as a complementary approach to the popular peptide mass fingerprinting methods^7^. These glycolipid barcodes are composites of relatively high molecular weight molecules that exhibit species-specific ions dependent on the unique glycolipid structures present in the membrane of the organism. Modified structures of these glycolipids result in ions that have been associated with resistance to the cationic antimicrobial peptide, colistin and are readily detectable by MALDI-TOF MS^7,15–20^. However, tools must be developed to rapidly translate these glycolipid barcodes into identifications.

In this study, we presented the development of prototype classifiers for the identification of *A. baumannii* and *K. pneumoniae* from glycolipid MALDI-TOF MS and described a flexible paradigm that is readily extendable to additional bacterial species. The feature engineering and machine learning methods that were employed resulted in classifiers capable of identifying *A. baumannii* and *K. pneumoniae* isolates and detecting colistin resistance in these species with high accuracy and specificity while maintaining high sensitivity from a glycolipid mass spectral dataset containing 48 other organisms. Though these classifiers do not identify individual strains, their ability to detect a target species and its colistin-resistant strains demonstrate the complementarity of this approach with current protein fingerprinting methods, which struggle to identify this form of antimicrobial resistance. Additionally, these types of classifiers are theoretically able to identify other antibiotic resistances, so long as the resistance mechanism is directly linked to lipid structures and resistant specimens are adequately represented in the training set.

Future methods to rapidly identify and characterize infections without culture, which is viable with our lipid-based approach, will need to demonstrate high performance on monomicrobial infections and polymicrobial infections alike. To test the suitability of our *A. baumannii* and *K. pneumoniae* classifiers on polymicrobial infections, we simulated 4,000 polymicrobial mixture mass spectra through the weighted averaging of real mass spectra from isolates in our previously reported glycolipid dataset. The species-level performance of the classifiers on the simulated polymicrobial mixtures was found to be consistently high, even when 5 species were represented in a mass spectrum. However, a noticeable hit to precision was observed for the detection of colistin resistance. Most notably, this is due to the false-positives that occur from the misclassification of mixtures containing colistin-susceptible isolates of the target species. We postulate that these misclassifications are the consequences of reliance on the few resistance-associated ions for each species. With increasing numbers of species represented in a mass spectrum, there is an increasing chance that one or more of these features is susceptible to interference from ions produced by a non-target organism.

As evidence for this theory, we investigated the most common component in false positive results when detecting colistin-resistant *A. baumannii* in the simulated mass spectra, *Salmonella minnesota*. Comparison between *S. minnesota* and colistin-resistant *A. baumannii* mass spectra revealed ions in the *S. minnesota* mass spectrum that confound the critical resistance-associated ion at feature *m/*z 2036.4575 (Supplementary Fig. S4). While a wide extraction window is currently necessary, as indicated by the variation of the extracted *m/z* for *A. baumannii* (Supplementary Fig. S4), enhancing the mass measurement accuracy and mass measurement precision of future libraries will likely aid in alleviating these interferences by allowing for narrower extraction windows. Along with enhanced mass accuracy, increased resolving power would also become valuable and decrease the smoothing needed for consistent feature extraction. Such increases in resolving power would also enable the use of isotopic information as features, as opposed to features that generally represent the average mass of ions. As a first practical improvement, future iterations of glycolipid datasets will employ internal mass calibrants, which will allow for the unbiased alignment of mass spectra, thereby improving mass measurement precision and accuracy and allowing for narrower feature extraction windows. While the current dataset consists of only intact lipid ions, the inclusion of fragmentation data, such as is already commonly collected in clinics on LC-MS/MS instruments, holds the potential to greatly increase the performance of these identifications.

As a final test of our *A. baumannii* and *K. pneumoniae* classifiers, we generated controlled, *in vitro* polymicrobial samples to represent mixtures containing the most common causative pathogen of UTIs, *E. coli*. These spike-in samples revealed that the classifiers were capable of reliably detecting their target species and colistin resistance in real mixture mass spectra. While these two-species mixtures represent a small set of many possibilities, they provide a promising glimpse into future classifiers specifically built for the purpose of pathogen identification from polymicrobial samples. However, to reliably create such classifiers for clinical applications, large datasets containing real polymicrobial infections will need to be collected to train classifiers specifically for this purpose. Additionally, classifiers trained on datasets that characterize specific polymicrobial mixtures may be useful for identifying secondary pathogens in an infection where the primary pathogen is known.

In conclusion, this study presents an extendable machine learning strategy for the identification of *A. baumannii* and *K. pneumoniae* and detection of colistin resistance in these species. Furthermore, we demonstrated the potential to identify these organisms from polymicrobial mixtures using simulated mass spectra and *in vitro* models of UTIs. With the success of these two prototypical pathogens and as the glycolipid mass spectral library continues to grow, we aim to rapidly expand these methods to other organisms in the future—even those with uncharacterized lipid A structures. As we continue to streamline the glycolipid extraction protocol and improve the limit of detection, the application of machine learning to the characterization of pathogens from glycolipid mass spectra will offer a complementary approach to the tool belt of clinical labs.

## Methods

### The isolate glycolipid mass spectra dataset

The isolate glycolipid mass spectra dataset used in this study was originally described in Leung *et al*., where full details can be found for strain selection, glycolipid extraction, and MALDI-TOF MS acquisition^7^. Briefly, colistin-susceptible strains of *A. baumannii* and *K. pneumoniae* were defined as having a minimum inhibitory concentration (MIC) ≤2 µg/mL colistin, whereas colistin-resistant strains were defined by an MIC ≥4 µg/mL colistin as recommended by the Clinical and Laboratory Standards Institute^29^. Lipid A and other membrane glycolipids were harvested from 1–5 mL overnight liquid cultures using a small-scale hot ammonium isobutyrate extraction protocol originally described by El Hamidi *et al*.^30^. Membrane lipid extracts were washed twice with methanol and resuspended in 2:1:0.25 chloroform/methanol/water (Fisher Scientific, Waltham MA; Quality Biological, Gaithersburg MD). Aliquots of 1 µL were manually spotted on stainless steel target plates with norharmane matrix (10 mg/mL in 2:1 v/v chloroform/methanol) (Sigma-Aldrich, St. Louis MO). Mass spectra were acquired as the sum of 900–1,000 laser shots on a Bruker Microflex LRF MALDI-TOF MS operated in negative ion and reflectron modes (Bruker Daltonics Inc., Billerica MA). The analyses were acquired using the equipped 337 nm nitrogen laser at 39.5% global intensity. The resolution and mass accuracy of the mass spectra in the dataset were estimated using the M + 1 molecular ion of the *m/z* 1910 structure in the 657* A. baumannii* mass spectra. By this method, the resolution was estimated to be 2,300 ± 200 *m*/Δ*m* at FWHM and the average mass error was found to be 0.73 ± 0.05 Da. The indicated uncertainties are 95% confidence intervals.

### Spectral processing and feature engineering

Mass spectra were converted to mzXML file format using msconvert (v3.0.9393, ProteoWizard). All analyses for this publication were performed in the R statistical programming language (v3.4.0)^31^. Spectral processing was performed using the MALDIquant (v1.16.2) and MALDIquantForeign (v0.10) R packages^32,33^. The mass spectra were square root-transformed and smoothed using a 161-point Savitzky-Golay filter^34^. The large smoothing window avoided inconsistencies in peak picking and extraction caused by variations in isotopic resolution between mass spectra. The mass spectra were baseline-corrected using the SNIP method over 60 iterations^35^.

Prior to import, the isolate mass spectra dataset was divided into training (60%) and test sets (40%). For feature selection and model training, only the training set was used. Features for the *A. baumannii* and *K. pneumoniae* classifiers were defined as the maximum intensity within ±1.5 *m/z* windows centered on the top 50 most intense molecular ions for the respective species. This window was selected after manual inspection of feature extraction windows for known lipid A structures. The intensities for each feature were normalized to the most intense extracted feature, resulting in features with values inclusively between 0 and 1. This normalization process accounted for differences in intensity due to factors such as differences in total analyte abundance. After feature selection with the training set, all further data were subjected to the same preprocessing and feature extraction, resulting in 50 features for use in classifier training and prediction.

### Machine Learning

Gradient boosted tree models, using the XGBoost algorithm, were chosen due to their ability to perform highly accurate classification and efficient training^25^. The xgboost (v0.6-4) R package was used for implementation of the XGBoost algorithm. Classifier training was performed using the isolate mass spectra training set. For *A. baumannii* and *K. pneumoniae*, 3-class models were trained to recognize the presence of the colistin-resistant or -susceptible target organism, or neither, by minimizing the multiclass logarithmic loss (*metrics* = *“mlogloss”)*. Rough parameter tuning was performed by grid search, optimizing the *max_tree_depth*, *min_child_weight*, and *gamma* at *eta* = *0.3* using 10-fold cross-validation. Final model parameters and the optimal number of iterations were selected by reducing *eta* to 0.01 and using 10-fold cross-validation.

Performance assessment of the final classifiers was performed using the PRROC (v1.3) R package for PR and ROC curve analysis and the caret (v6.0-76) R package for other statistics^36,37^. To translate the 3-class model scores to the metrics investigated, one-vs-all analysis of the scores was performed. To investigate a species-level identification, the *species* score is the sum of the *colistin-resistant* and *colistin-susceptible* scores, which is compared against the *other species* score for the classifier. Alternatively, the *colistin-resistant* score is compared against the sum of the *colistin-susceptible* and *other species* scores for a classifier. Unless otherwise noted, the error bars for all performance metrics indicate the 95% confidence intervals as calculated empirically using 2,000 bootstrapped replicates.

### Simulation of polymicrobial mass spectra

For the simulation of polymicrobial mass spectra, the entire isolate mass spectra dataset was used. Species to be included in a spectrum were chosen at random with a fixed probability of 0.3/*n* of the spectrum containing an *A. baumannii* or *K. pneumoniae* spectrum, where *n* is the number of species in the spectrum. Other species in a spectrum each had an equal chance of being observed, without replacement. A mass spectrum for each selected species was then chosen at random to represent the species in the final polymicrobial mass spectrum. With the mass spectra chosen, the intensities of each spectrum were multiplied by a weight randomly drawn from the set of 1, 0.5, 0.25, and 0.1, with replacement, under the restraint that at least one spectrum had a weight of 1. The weighted mass spectra were then averaged to yield a simulated polymicrobial spectrum. This simulation strategy is similar to the approach presented by Mahé *et al*., and results in a linear combination of isolate-species mass spectra to build a polymicrobial mass spectrum^38^. This was repeated 1,000 times each for 1–5 species per mass spectrum, resulting in 4,000 simulated polymicrobial mass spectra and 1,000 simulated isolate mass spectra.

### Generation of UTI spike-in models and mass spectra dataset

Bacterial species were selected based on their prevalence in UTIs. *E. coli* (ATCC 25922), the most common causative agent, followed by *K. pneumoniae* (A2, colistin-susceptible and A5 colistin-resistant strains) and *A. baumannii* (SM1646 colistin-susceptible and PM3757 colistin-resistant strains) were selected for UTI model generation^27^. Colonies of *E. coli* and colistin-susceptible strains were picked from agar plates and then inoculated in Luria-Bertani (LB) medium for overnight culture. Colistin-resistant strains were cultured overnight in LB medium with 2 μg/mL colistin sulfate to prevent contamination from other unwanted species. Overnight liquid cultures were enumerated and aliquoted for lipid A isolation.

Prior to lipid A extraction, these 5 strains pellets were resuspended in 1 mL sterile urine individually. *E. coli* was mixed with antibiotic-susceptible or -resistant *A. baumannii* or *K. pneumoniae* strains at various volumetric ratios. The total mixture (1 mL) was spiked into sterile urine that had been pre-warmed to 37 °C to mimic infection conditions. Bacterial spiked urine samples were well vortexed and incubated in a warm-room for 5 minutes. The same lipid A micro-extraction method that is briefly described in the isolate glycolipid mass spectra dataset section was used for extraction. Aliquots of 0.75 μL lipid A extracts were manually spotted on a MALDI target plate with the same volume of norharmane matrix solution.

Mass spectra were acquired in negative ion mode using a Bruker Microflex LRF MALDI-TOF MS (Bruker Daltonics Inc., Billerica MA) operated in reflectron mode. The instrument was calibrated with Agilent Tuning Mix (Agilent Technologies, Santa Clara, CA). Each sample was acquired at 68% laser power with 900 laser shots summed and performed in triplicate.

## Electronic supplementary material

Supplementary Information

Supplementary Dataset 1

Supplementary Dataset 2

Supplementary Dataset 3

## Data Availability

All data used for this publication is freely available through the University of Maryland, Baltimore Office of Technology Transfer. All R code needed to fully reproduce this analysis and a link to the data are available at https://github.com/wfondrie/DetectingColistinResistance.
