# Supplementary material for: Pathogen Identification Direct From Polymicrobial Specimens Using Membrane Glycolipids
Source: Sci Rep. 2018 Oct 26;8:15857. doi: 10.1038/s41598-018-33681-8 (PMC6203844; doi:10.1038/s41598-018-33681-8)
Supplement: Supplementary file 1 — Supplementary Information [file 41598_2018_33681_MOESM1_ESM.pdf]

Pathogen Identification Direct From Polymicrobial Specimens Using Membrane Glycolipids – Supplementary Information

William E. Fondrie[1], Tao Liang[2], Benjamin L. Oyler[3], Lisa M. Leung[4,5], Robert K. Ernst[4], Dudley K. Strickland[1,6,7] and David R. Goodlett[2,*]

[1]Center for Vascular and Inflammatory Diseases, University of Maryland School of Medicine, Baltimore, MD, 21201, USA;

[2]Department of Pharmaceutical Sciences, University of Maryland School of Pharmacy, Baltimore, MD, 21201;

[3]Toxicology and Pharmacology, University of Maryland School of Medicine, Baltimore, MD, 21201, USA;

[4]Department of Microbial Pathogenesis, University of Maryland School of Dentistry, Baltimore, MD, 21201, USA;

[5]Divisions of Microbiology and Molecular Biology, Laboratories Administration, Maryland Department of Health, Baltimore, Maryland, 21205, USA (Current Affiliation);

[6]Department of Surgery; University of Maryland School of Medicine, Baltimore, MD, 21201, USA;

[7]Department of Physiology; University of Maryland School of Medicine, Baltimore, MD, 21201, USA;

***Corresponding Author**
Email Address: dgoodlett@rx.umaryland.edu
Address: School of Pharmacy
University of Maryland
Pharmacy Hall North, Room 623
20 N. Pine Street
Baltimore, MD, 21201

SUPPLEMENTARY FIGURES

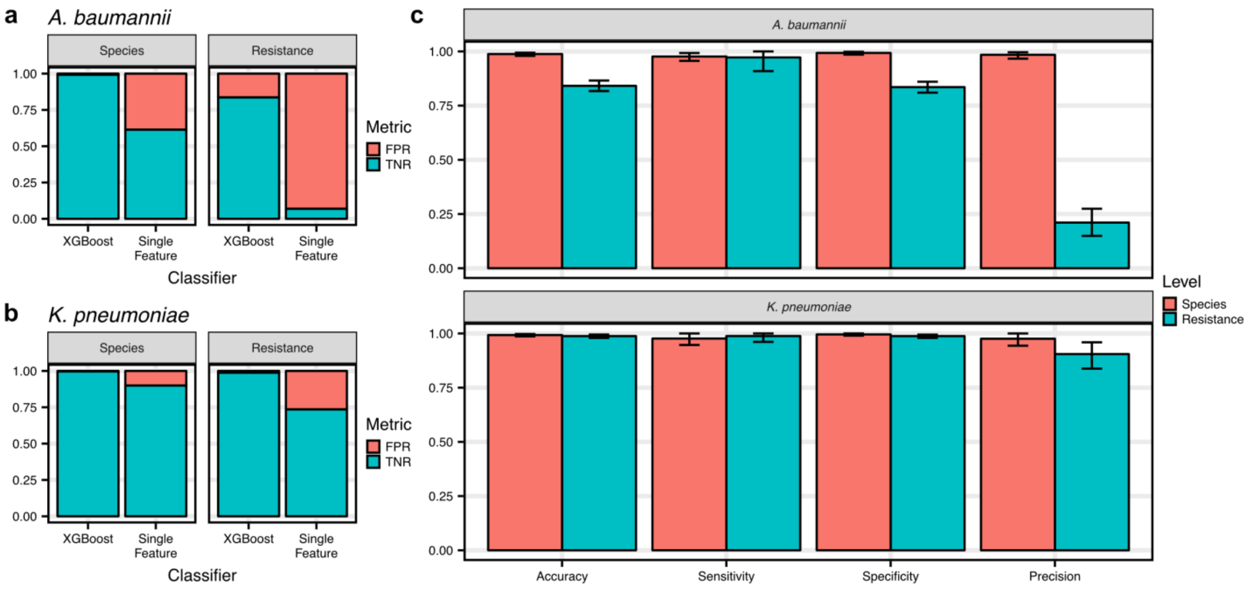

Supplementary Figure S1. The classifiers demonstrate high performance at a 97% threshold. The false positive rate (FPR) and true negative rate (TNR) of the XGBoost and Single Feature classifiers at 97% sensitivity for **(a)** *A. baumannii* and **(b)** *K. pneumoniae*. The XGBoost classifiers give a markedly lower FPR than the Single Feature baseline classifier. **(c)** High performance is observed across a variety of performance metrics for the XGBoost classifiers. Error bars indicate 95% confidence intervals.

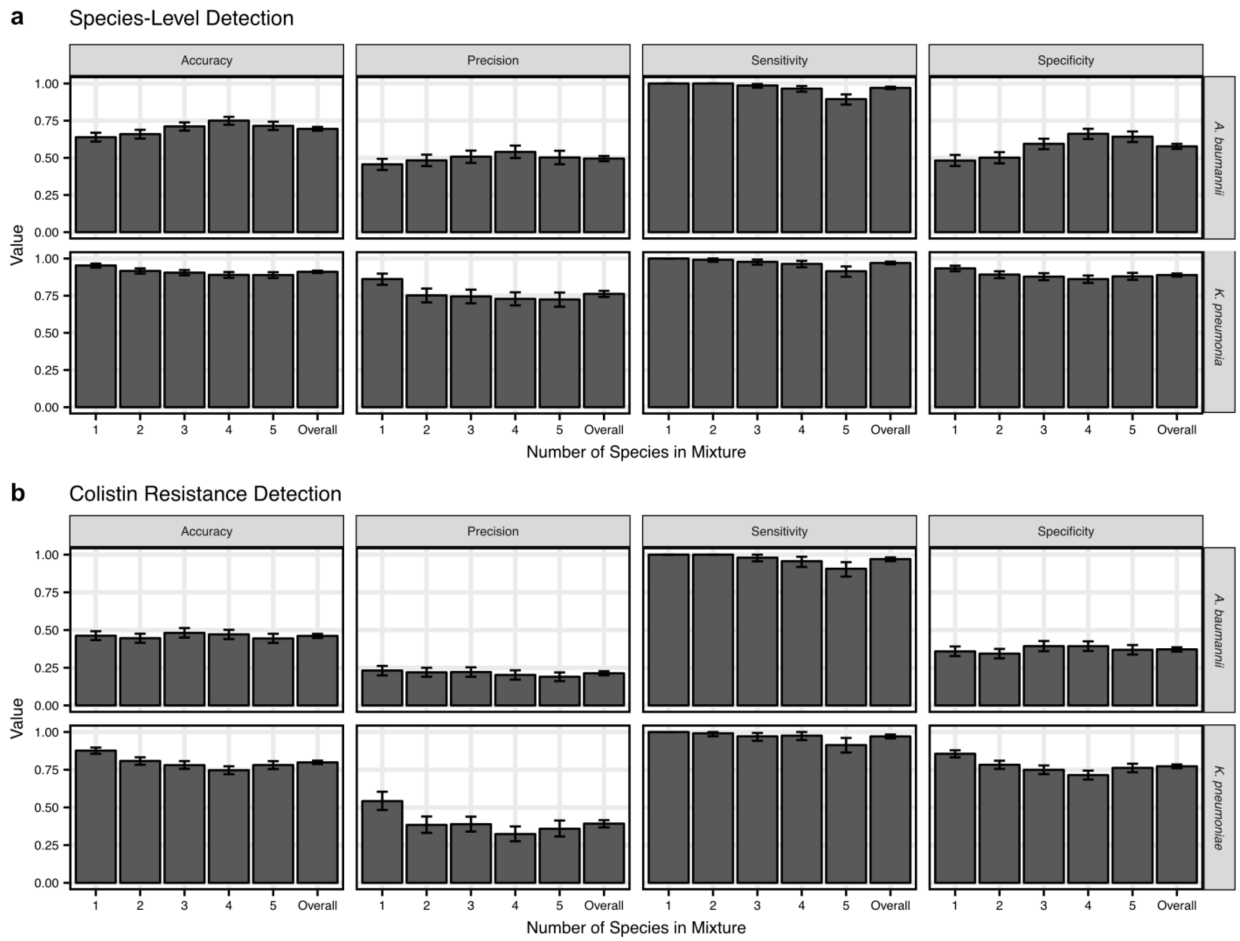

Supplementary Figure S2. The classifiers perform well on the simulated polymicrobial mass spectra. Performances of the classifiers were evaluated at a threshold that maintains 97% sensitivity for the overall evaluation of the 5,000 simulated mass spectra. **(a)** Species-level performance at detecting *A. baumannii* and *K. pneumoniae* indicates that, while less reliable than from isolate spectra, the classifiers can detect *A. baumannii* and *K. pneumoniae* species from polymicrobial mixtures. **(b)** The detection of colistin resistance from the simulated polymicrobial spectra appears to decrease with the complexity of the mass spectra at the stringent 97% overall sensitivity threshold. Error bars indicate 95% confidence intervals.

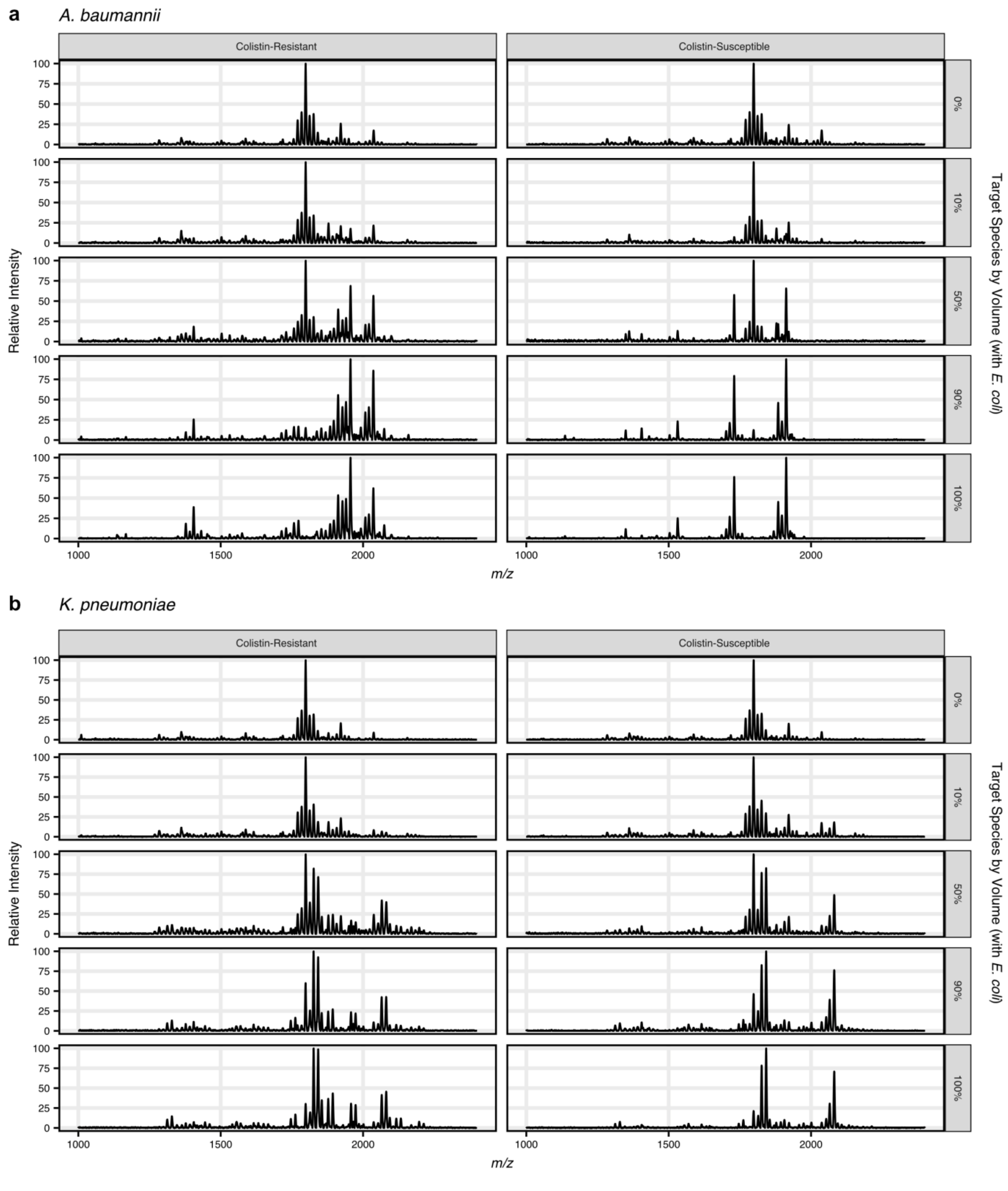

Supplementary Figure S3. The ratiometric emergence of species-specific ions are readily observable in the MALDI-TOF mass spectra from *in vitro* UTI mixtures. (a) Though ions from *E. coli* generally occupy similar *m/z* space as ions from the target organisms, signature ions for *A. baumannii* (*m/z* 1910 and resistance ion *m/z* 2033) can be seen with ratio-dependent intensities in the mixture mass spectra. **(b)** Similarly, signature ions for *K. pneumoniae* (*m/z* 1824 and 1840, as well as resistance ions *m/z* 1955 and 1971) observed to increase with increasing proportions of *K. pneumoniae* in the sample.

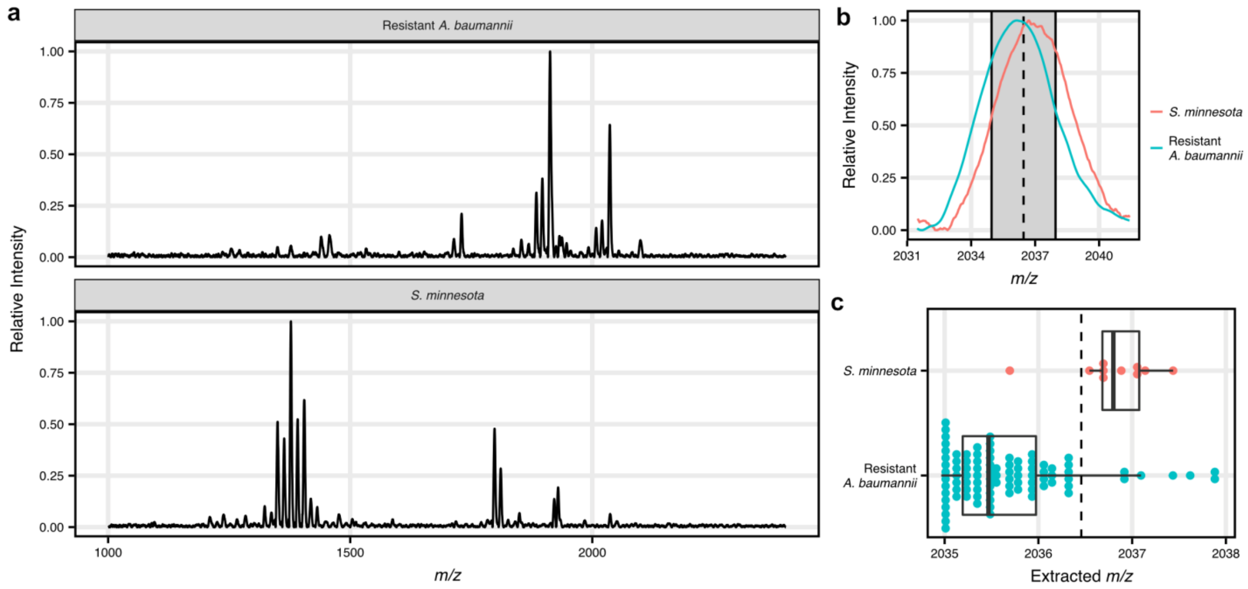

Supplementary Figure S4. Low precision observed with the simulated mixtures may be improved with increased mass accuracy and resolving power. (a) Representative spectra for colistin-resistant *A. baumannii* and the most common component in the false positive simulated mixtures, *S. minnesota*, reveal ions in shared *m/z* space as the important *A. baumannii* resistance-associated ion at feature *m/z* 2036.4575. **(b)** Close inspection of the feature extraction window (shaded box) for these spectra reveal ions that would be indistinguishable using the current feature extraction parameters due to the current mass spectral resolution, post-processing. **(c)** Investigation of the measured *m/z* of extracted maximum intensity for feature *m/z* 2036.4575 shows that mass accuracy is a limiting factor. True resistance-associated ions are extracted across the full 3 *m/z* window, which is also occupied by ions from *S. minnesota.*
